# The complete mitochondrial genome of *Apis mellifera jemenitica* (Insecta: Hymenoptera: Apidae), the Arabian honey bee

**DOI:** 10.1080/23802359.2020.1717383

**Published:** 2020-01-27

**Authors:** Leigh Boardman, Amin Eimanifar, Rebecca T. Kimball, Edward L. Braun, Stefan Fuchs, Bernd Grünewald, James D. Ellis

**Affiliations:** a Honey Bee Research and Extension Laboratory, Entomology and Nematology Department, University of Florida, Gainesville, FL, USA;; b Department of Biology, University of Florida, Gainesville, FL, USA;; c Institut für Bienenkunde, Goethe-Universität Frankfurt am Main, Oberursel, Germany

**Keywords:** Mitogenome, next-generation sequencing, *Apis mellifera yemenitica*, *Apis mellifera nubica*

## Abstract

The mitochondrial genome of a worker *Apis mellifera jemenitica* was 16,623 bp. It consisted of 13 protein-coding genes, 22 transfer RNAs, two ribosomal RNAs and a control region. Phylogenetic analyses suggest a close relationship between *A. m. jemenitica*, *A. m. lamarckii* and *A. m. syriaca*.

The Arabian honey bee, *Apis mellifera jemenitica* (Ruttner, 1976, junior synonym: *nubica*; often written *yemenitica*), is the only *Apis mellifera* subspecies to occur naturally in both Africa and Asia with a distribution from the Arabian peninsula in the East, to south of the Sahara in the West (Al-Ghamdi et al. 2013). Previous studies on the mitochondrial DNA of *A. m. jemenitica* are not in agreement as to which *A. mellifera* lineage this subspecies belongs (e.g. Franck et al. 2001; El-Niweiri and Moritz 2008; reviewed in Al-Ghamdi et al. 2013). Here, we present the mitochondrial genome (GenBank: MN714161) of a worker *A. m. jemenitica* honey bee (Voucher No. 1544, Ruttner Bee Collection at the Bee Research Institute at Oberursel, Germany). This sample was collected in 1988 by Prof. H. Hoppe in Bani Shiab, Yemen (15°29 N, 43°44 E), and subspecies identity was confirmed morphometrically.

Genomic DNA was extracted and prepared for PE-150 bp sequencing on Illumina Hi-Seq 3000/4000 (San Diego, CA) following the method described by Eimanifar et al. (2017). The quality of the resulting data was assessed using FastQC (Andrews 2010), and trimmed with Trimmomatic (Bolger et al. 2014). Trimmed data were mapped in Geneious Prime 2019.0.4 (Kearse et al. 2012) using the method from Boardman et al. (2019) and the mitogenome of *A. m. lamarckii* (KY464958) as a reference. Mitos2 was used to annotate the assembled mitogenome (Bernt et al. 2013) before it was manually adjusted to match the *A. m. capensis* annotation (KX870183) in Geneious Prime. The 13 protein-coding genes (PCGs) and two ribosomal RNA (rRNA) sequences were manually aligned to sequences from other *Apis* mitogenomes in Mesquite v3.5 (Maddison and Maddison 2018). For phylogenetic estimation, we used RAxML 8.2.10 (Stamatakis 2014) with the GTRGAMMA model and 1000 bootstrap replicates (-f a option) on the CIPRES Science Gateway V.3.3 (Miller et al. 2010), and *p*-distances were obtained using PAUP 4.0a (Swofford 2003).

The complete mitochondrial genome of *A. m. jemenitica* was 16,623 bp long. It was AT-rich, consisting of 43.4% A, 41.5% T, 9.5% C, and 5.5% G. Of the 13 PCGs, nine were located on the light strand and four on the heavy strand. Four start codons were used: ATT (*atp8*, *co2*, *nad1*, *nad4l*, *nad5*, *nad6*), ATG (*atp6*, *co3*, *cytb*, *nad4*), ATA (*co1*, *nad3*), and ATC (*nad2*). All 13 PCGs used TAA as the stop codon. *Atp8* and *atp6* overlapped, sharing 19 bp. Of the 22 transfer RNAs (tRNAs), the shortest was tRNA-Gln with 63 bp and the longest at 80 bp was tRNA-Thr. The two rRNAs were both AT-rich and located on the heavy strand (16S: 1,358 bp, 84.6% AT; 12S: 788 bp, 81.5% AT).

Previous samples of *A. m. jemenitica* from Yemen had both O- and A-lineage haplotypes (Smith, unpublished, in Al-Ghamdi et al. 2013). Here, our sample was closest to *A. m. lamarckii* (*p* = 0.00381) and *A. m. syriaca* (*p* = 0.00586) (Figure 1), suggesting it forms part of the Z subgroup of the A-lineage (Alburaki et al. 2011). Sequencing additional samples of *A. m. jemenitica* across its geographic distribution would be informative.

**Figure 1.**
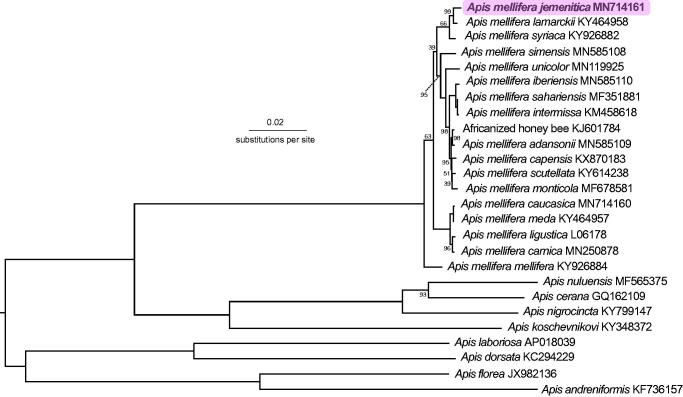
Phylogenetic tree showing the relationship between *Apis mellifera jemenitica* (GenBank: MN714161) and 25 other *Apis* honey bee mitogenomes. The tree is midpoint rooted, node labels indicate bootstrap values, and unlabeled lineages are 100%.
